# Carbon-Based Nanomaterials in Sensors for Food Safety

**DOI:** 10.3390/nano9091330

**Published:** 2019-09-17

**Authors:** Mingfei Pan, Zongjia Yin, Kaixin Liu, Xiaoling Du, Huilin Liu, Shuo Wang

**Affiliations:** 1 State Key Laboratory of Food Nutrition and Safety, Tianjin University of Science & Technology, Tianjin 300457, Chinayinzongjiasiss@126.com (Z.Y.); lkx13642168374@163.com (K.L.); duxiaoling98@163.com (X.D.); 2 Key Laboratory of Food Nutrition and Safety, Ministry of Education of China, Tianjin University of Science and Technology, Tianjin 300457, China; 3 College of Food and Health, Beijing Technology and Business University, Beijing 100048, China; liuhuilin@btbu.edu.cn

**Keywords:** carbon-based nanomaterials, chemo- and biosensor, food safety

## Abstract

Food safety is one of the most important and widespread research topics worldwide. The development of relevant analytical methods or devices for detection of unsafe factors in foods is necessary to ensure food safety and an important aspect of the studies of food safety. In recent years, developing high-performance sensors used for food safety analysis has made remarkable progress. The combination of carbon-based nanomaterials with excellent properties is a specific type of sensor for enhancing the signal conversion and thus improving detection accuracy and sensitivity, thus reaching unprecedented levels and having good application potential. This review describes the roles and contributions of typical carbon-based nanomaterials, such as mesoporous carbon, single- or multi-walled carbon nanotubes, graphene and carbon quantum dots, in the construction and performance improvement of various chemo- and biosensors for various signals. Additionally, this review focuses on the progress of applications of this type of sensor in food safety inspection, especially for the analysis and detection of all types of toxic and harmful substances in foods.

## 1. Introduction

Food safety is usually defined as the scientific discipline that describes the preparation, treatment and storage of food products in ways which can prevent foodborne illness. In recent years, food safety and quality have received widespread attention [1,2]. Food insecurity, such as pesticide residues, illegal additives, allergens, pathogens and other unsafe factors, not only seriously affects people’s health, but also limits the rapid development of the food industry to a certain extent [3,4]. The development of analytical methods or equipment that meet the requirements of modern detection of various hazardous substances in foods is an important and crucial aspect of food safety studies. Due to the complex matrix of food samples and the presence of trace amounts of hazardous agents, high-throughput, low-cost, accurate, sensitive and convenient analytical methods or devices are becoming the mainstream of food safety testing [5,6,7]. A sensor composed of an identification element and a signal transducer characterized by simple structure, high portability and low price can compensate for disadvantages of expensive and universal popularity of the existing instrumental methods [8,9,10]. Such a sensor may be suitable for on-site and real-time qualitative and quantitative analysis of harmful substances in foods and thus inhabit a wider research and development space. In recent studies, various chemical or biological sensing devices based on various working principles have been developed for the detection of various hazardous substances in foods, thus becoming a focus of research in the field of food safety [11,12].

Carbon-based nanomaterials have attracted considerable interest of scientists since their discovery. According to their spatial dimensions, carbon-based nanomaterials can be roughly divided into fullerenes (zero-dimensional), carbon nanotubes (one-dimensional), graphene (two-dimensional), graphene coil (multidimensional), etc. [13,14]. In Table 1, a variety of properties of the carbon nanomaterials were presented.

In recent years, numerous studies on the preparation, modification or application of carbon-based nanomaterials have been actively published. Carbon-based nanomaterials of various morphologies (needle, rod, barrel, etc.) have been prepared and successfully applied in various research areas [28,29,30]. Generally, the heterocyclic state of the C-C bonds in the carbon-based nanomaterials determines their unique spatial structure resulting in the remarkable chemical and electronic properties. Characteristic features of the carbon-based nanomaterials include small-size, interface, surface, dielectric confinement, macroscopic quantum tunneling effects, etc.; their advantages include ease of preparation, stability and high heat and electronic conductivity [15,16]. These merits promote wide use of this type of nanomaterial in several areas, including environmental monitoring, energy storage, life science, etc. [31,32]. Carbon-based nanomaterials have been used in the development of high-performance sensing devices for food safety inspection to produce, identify and enhance the sensing signals. In particular, in-depth studies of new carbon materials, such as graphene and carbon dots, enhanced the potential of carbon-based sensors and their application prospects in the development of food safety inspection devices characterized by high precision, high protection from interference, and convenience [33,34,35].

This paper reviews various characteristics of carbon-based nanomaterials and their relevant applications in food safety inspection. The latest studies on the fabrication and construction of new high-performance sensing devices for food safety detection are introduced in special detail. This paper summarizes the status of research and development trends of chemo- and biosensors based on carbon-based nanomaterials used in the detection and analysis of residual pesticides, veterinary drug, illegal food additives, allergens and other major toxic and harmful substances, thus promoting the further study of carbon-based nanomaterials, especially in developing new types of high performance sensing devices to meet the requirements of food safety detection and to improve the detection levels with certain theoretical guidance.

## 2. Carbon-Based Nanomaterials

### 2.1. Ordered Mesoporous Carbon (OMC)

Mesoporous carbon materials (diameter between 2 and 50 nm) are a new type of non-silica mesoporous materials that were discovered and have attracted considerable attention in recent years [17,18]. Compared with mesoporous silicon materials, mesoporous carbon materials have several special excellent properties, such as high specific surface area and porosity, adjustable pore size, controllable pore wall composition and structure, simple synthesis, and a lack of physiological toxicity. At the same time, high thermal and hydrothermal stability and extremely large specific surface area and pore volume can be obtained by optimizing and controlling the synthesis conditions, thus making this type of material very promising for a wide range of applications, including in adsorbent carriers [20,36], catalyst supports [37,38,39], hydrogen storage materials [40,41,42], and electrode materials [43,44,45].

**Figure 1:**
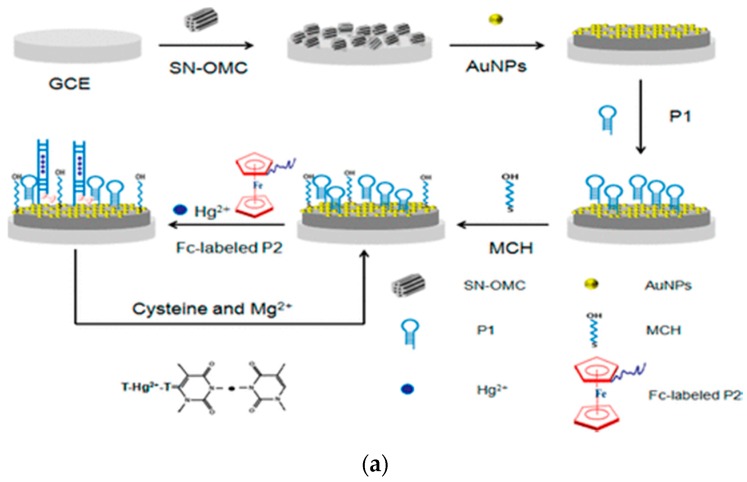

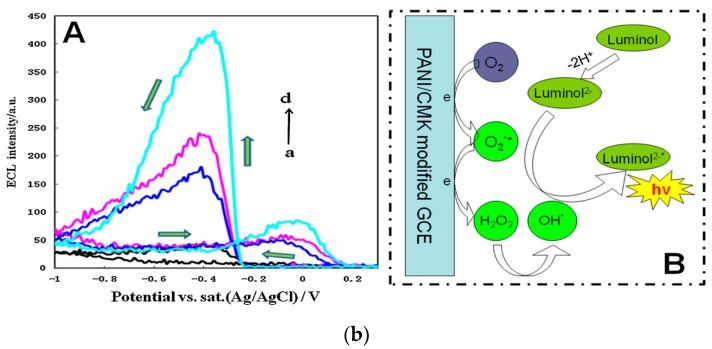
Application of OMC nanomaterials in the fabrication of sensors. (**a**) Assembly diagram of electrochemical aptasensor based on the OMC nanomaterials for Hg^2+^ detection. Reproduced with permission from reference [61]. Copyright American Chemical Society, 2018; (**b**) modification of OMC nanomaterials to enhance the conductivity and stability of sensors. (**b**-**A**) The ECL behavior of luminol at PANI/CMK/GCE in PBS solution; (**b**-**B**) the process of luminol react with the ROSs. Reproduced with permission from reference [62]. Copyright Elsevier, 2012.

Mesoporous carbon materials can be divided into disordered mesoporous carbon and OMC based on the regularity of pores [46]. Disordered mesoporous carbon is usually obtained by the catalytic activation of metal ions [47], carbonization of polymers, and carbonitriding or oxidation of silica templates by organic aerogels, resulting in lower regularity and uniformity of the pore structure [48,49]. Thus, the disordered carbon materials can be applied as an excellent anode for sodium ion exchange batteries and other energy storage devices [50,51,52]. Compared with disordered mesoporous carbon, OMC materials are composed of highly ordered and macroporous carbon nanorods [53,54] that have better electrochemical stability and unique properties that other materials do not possess, such as a highly ordered pore structure, an easily controlled mesoporous structure, narrow pore size distribution, and larger specific surface area (2000 m^2^ g^−1^) and specific pore volume (1.5 cm^3^ g^−1^) [19,20]. The synthesis of OMC is usually performed by the hard template method using mesoporous silica molecular sieves as a template, selecting a suitable precursor and carbonating the precursor in the pore of the mesoporous template with subsequent etching of the mesoporous silica template using NaOH or HF solutions [55,56]. Mesoporous carbon materials have a wide range of applications in material synthesis [57], catalyst carrier, adsorption separation [42,58,59], and electronic devices [60]. Cui and coworkers fabricated a novel aptasensor using a sulfur nitrogen codoped OMC (SN-OMC) and thymine-Hg^2+^-thymine mismatch structure, which has a fine linear correlation for Hg^2+^ (0.001–1000 nM) with a detection limit (LOD) of 0.45 pM [61] (Figure 1a).

OMC nanomaterials have excellent electrochemical capacitance performance and have become an ideal material for electrochemical capacitors [63]. Dai et al. constructed a highly porous three-dimensional sensing interface on a glassy carbon electrode (GCE) using OMC and polyaniline. This polyaniline/OMC composite-modified electrode is an efficient electrochemiluminescence platform for luminol due to the attractive features of excellent electrical conductivity, extremely well-ordered pore structure and high specific pore volume. Electrolyte ions can freely migrate in the regular pore of mesoporous carbon to rapidly form an electric double layer and weaken the dispersion effect of the capacitor resulting in strong charge-discharge capacity (Figure 1b). Pharmacologically, ractopamine (RAC) is a TAAR1 and β-adrenoreceptor agonist that stimulates β1 and β2 adrenergic receptors. As a result, RAC is an illegal active growth-promoting ingredient in the products used in food animals, such as swine and cattle. Yang et al. constructed an electrochemical sensor using OMC for sensitive detection of toxic RAC in swine samples [64]. OMC-modified electrode showed remarkably enhanced electrocatalytic activity toward RAC oxidation with a great increase in electrochemical current to achieve favorable detection sensitivity and selectivity. Moreover, OMC has been combined with Prussian blue (PB) for signal enhancement. A three-dimensional molecularly imprinted electrochemical sensor was developed for ultra-sensitive and specific quantification of metolcarb (a carbamate pesticide). The introduced OMC material aimed to enhance the electrochemical response by improving the structure of the modified electrodes to facilitate the charge transfer of PB (inherent probe) [65]. 

### 2.2. Carbon Nanotubes (CNTs)

**Figure 2:**
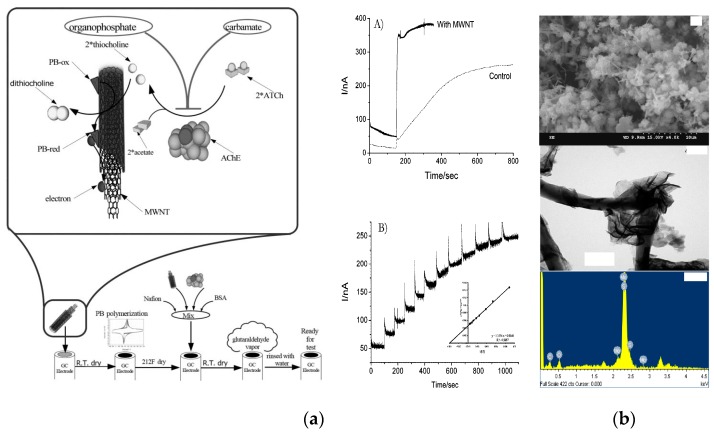
Significant performance of electrochemical sensors based on MWCNT materials. (**a**) AChE/PB/MWNT electrochemical sensor for pesticide detection. Reproduced with permission from reference [81]. Copyright Royal Society of Chemistry, 2008. (**b**) Characterization of MoS_2_/MWCNTs nanocomposite: SEM, TEM and EDX. Reproduced with permission from reference [82]. Copyright Elsevier, 2017.

CNTs are hollow tubular one-dimensional nanomaterials composed of hexagonal carbon atoms identified for the first time by Iijima in 1991 [66,67]. Because of their unique spatial structure, physical and chemical properties, and simple preparation methods, CNTs have become one of the most widely studied carbon materials, and remarkable progress has been achieved in several research areas [68]. Usually, the main C atoms in CNTs have sp2 hybrid orbitals; when the spatial topology is formed, sp3 hybrid orbitals can be formed. A certain degree of bending is present between the grid structures, which are composed of hexagons. Due to the formation of the chemical bonds in the hybrid and due to overlapping, a highly delocalized π bond in the outer layer of CNTs becomes a chemical basis for its noncovalent binding to certain macromolecules such as proteins, nucleic acids and carbohydrates [69]. Depending on the arrangement of their graphene cylinders, CNTs can be divided into single-walled CNTs (SWCNTs) and multiwalled CNTs (MWCNTs). In general, SWCNTs with high chemical inertness are relatively simple and have a defect-free structure and surface, while MWCNTs often have small hole-like defects, which can be easily captured between the layers during their initial formation making the chemical properties of MWCNTs extremely active. Various electrochemical properties of SWCNTs and MWCNTs, such as catalytic activity [70,71], stability [72,73], electrical conductivity [74,75] and biocompatibility [76,77,78], have very important applications in the construction of chemical or biological sensors for food safety [79,80]. Chen et al. used MWCNTs to develop an acetylcholinesterase (AChE)-based electrochemical sensor for a sensitive and cost-effective pesticide assay in environmental and food samples [81] (Figure 2a). The MWCNTs were designed to play dual enhancement roles. The first role is to significantly increase the surface area, facilitating the electrochemical polymerization of PB; the second role involves the effective maintenance of the enzymatic activity of AChE decreasing Michaelis-Menten constant (*K*_m_). The developed MWCNT-based electrochemical sensor exhibited stable, reproducible and rapid response towards a series of pesticides in real samples. 

A hybrid material that consists of molybdenum disulfide nanosheet (MoS_2_) coating of the MWCNT surface was prepared for the determination of chloramphenicol (CAP), a broad-spectrum antibiotic acting by interfering with bacterial protein synthesis [82] (Figure 2b). The MoS_2_/MWCNT nanocomposite had great electrochemical property and displayed remarkable catalytic ability to CAP. The MoS_2_/MWCNT-modified electrode responded linearly in the CAP concentration range from 0.08 to 1392 μM and achieved a low LOD of 0.01502 μM. 

CNT materials with good catalytic activity and conductivity greatly reduce overpotential and efficiently accelerate the electron transfer in electrochemical reactions. Compared with ordinary materials, a sensor with CNTs as modifiers usually has great sensitivity, wide linear detection range and fast response [83,84]. Bhardwaj and coauthors utilized the Ab-SWCNT bioconjugates to develop a convenient, low-cost paper-based electrochemical immunosensor for label-free detection of *S. aureus* [85]. The anti-*S. aureus* Abs were covalently attached onto SWCNTs and immobilized on the working electrode surface to recognize the analyte, causing the changes of peak current. This remarkable sensor showed a good linearity (R^−2^ = 0.976) between an increase of peak current and logarithm of *S. aureus* concentration (10–10^7^ CFU mL^−1^) with less time (30 min) and a limit of detection of 13 CFU mL^−1^ in milk, indicating high sensitivity of the immunosensor. A multijunction sensor was designed by Kara et al. using SWCNT for multiplexed detection of foodborne pathogens [86]. The SWCNTs and polyethylenimine were coated on gold tungsten wires and formed a 2 × 2 junction array functionalized with streptavidin and biotinylated Abs. The introduction of SWCNTs aimed to reduce the background noise and to emphasize the response of biorecognition reactions between Ab and Ag. A MWCNTs/sol-gel-derived silica/chitosan nanobiocomposite was used to immobilize cholesterol esterase (ChEt) and cholesterol oxidase (ChOx) onto indium-tin-oxide (ITO) glass [87]. This new nanobiocomposite maintains the activity and stability of ChEt and improves the sensitivity (3.802 μA mM^−1^) while reducing the response time to 1002 s. Parveen et al. developed a fiber-optic probe coated by silver and CNT/copper nanoparticle (CuNPs) nanocomposite for nitrate sensing [88]. The target nitrate was reduced during interaction with CuNPs and formed NH_4_^+^ to change the dielectric properties of the CNT/CuNP nanocomposite, measured as a shift of resonance wavelength. 

**Figure 3:**
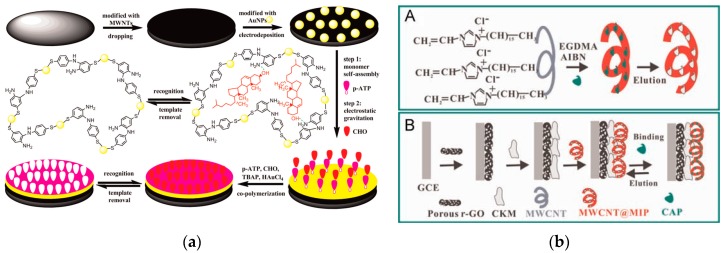
Application of MWNTs in molecularly imprinted biomimetic sensors. (**a**) The preparation procedure of AuNPs/MWNTs/GCE@MIP membrane. Reproduced with permission from reference [95]. Copyright 2015 Elsevier. (**b**) Scheme of the construction procedure of a MWCNTs@MIP-CAP-based sensor. Reproduced with permission from reference [96]. Copyright Elsevier, 2015.

Molecularly imprinted polymers (MIPs) have the binding sites for specific recognition of a template molecule allowing for specific recognition in complex and difficult environments [89,90]. Therefore, MIPs have been extensively studied in purification, separation and detection of matrices in food, medical or environmental samples in recent years [91,92]. Molecularly imprinted electrochemical sensor is a new type of biomimetic sensors that uses MIPs as a recognition element, having high sensitivity and selectivity, excellent stability, ease of preparation, low cost, miniaturization and easy automation [12,93,94]. An MIP electrochemical sensor for cholesterol detection was constructed on a GCE modified with MWCNTs and Au nanoparticles (AuNPs) [95] (Figure 3a). The MIP membrane was electropolymerized onto the electrode surface in a solution containing *p*-aminothiophenol, HAuCl_4_, tetrabutylammonium perchlorate and cholesterol. The Au-S bonds and hydrogen-bonding interactions were used to enhance the stability of sensor detection. The MWCNT material introduced into the molecular imprinting crosslinking system was used to overcome internal electron transport barriers and to further improve the detection sensitivity of molecularly imprinted biomimetic sensors. This feature is very important in the analysis of trace substances in the matrix of complex food products. Yang and coworkers synthesized 3-hexadecyl-1-vinylimidazolium chloride (C_16_VimC_l_) to improve the dispersion of MWCNTs, and to obtain MWCNTs@MIP of CAP on the MWCNT surface [96] (Figure 3b). Furthermore, the MWCNTs@MIP was applied as a coating on a mesoporous carbon and porous graphene (GO)-modified GCE to construct an electrochemical sensor that offers an excellent response to CAP and satisfactory results in real samples.

**Figure 4:**
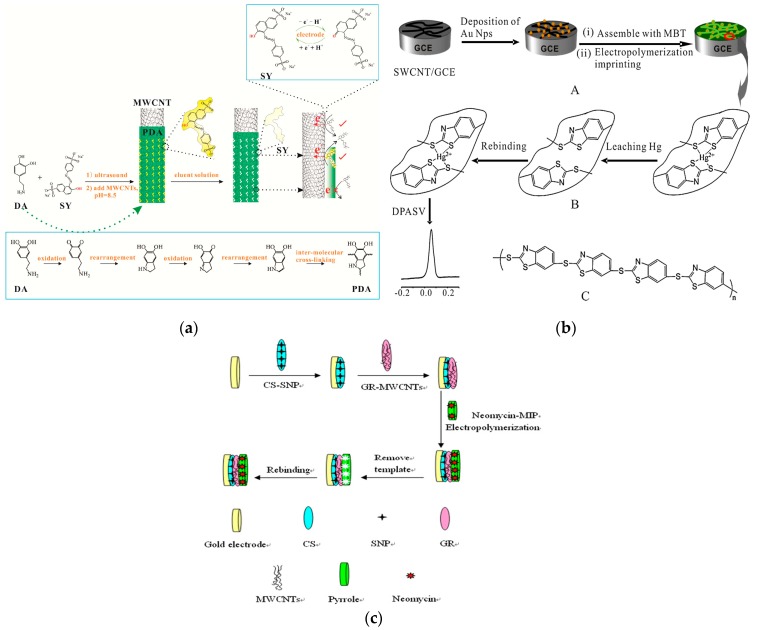
Application of CNTs in MIP-based sensors. (**a**) Fabrication of MWCNT@MIP-PDA sensor for sunset yellow. Reproduced with permission from reference [97]. Copyright 2018 Elsevier. (**b**) Schematic diagram of Hg(II)-imprinted PMBT/AuNPs/SWCNTs/GCE. Reproduced with permission from reference [102]. Copyright Elsevier, 2012. (**c**) Preparation of CS-SNP/graphene-MWCNTs composite-decorated gold electrode. Reproduced with permission from reference [103]. Copyright Elsevier, 2013.

Yin and Li synthesized polydopamine (PDA) by monomeric self-polymerization in water and used it to modify the surface of MWCNTs to prepare an MIP for sunset yellow [97]. The prepared imprinted electrochemical sensor showed remarkably selective and ultrasensitive response to the template. The improved behavior is caused by the highly matched imprinted cavities on the excellent electrocatalytic matrix of MWCNTs and the electronic barrier of the non-imprinted PDA. This study proposed a convenient and efficient imprinting strategy with great potential application value in designing other PDA-based MIP sensors. Other nanomaterials, such as metal NPs and transition metal complexes, can be efficiently modified on CNT surfaces to obtain composite nanomaterials, which can improve the detection performance of biomimetic sensors in food samples [98,99,100,101]. Fu et al. were the first to electropolymerize Hg^2+^ imprinting poly (2-mercaptobenzothiazole) films on the GCE surface modified by AuNPs and SWCNT nanohybrids for electrochemical detection of Hg^2+^ [102] (Figure 4a). Huang and coworkers successfully prepared novel chitosan-silver nanoparticle (CS-SNP)/graphene-MWCNTs composite-decorated Au electrode [103] (Figure 4b). The electropolymerized molecularly imprinted film of neomycin has high binding affinity and selectivity, and good reproducibility and stability in practical application. Pan et al. used MWCNTs and Salen-Co(III) to sensitize a new MIP for the recognition element of a sensor for methimazole determination [104]. This is the first report of using MWCNTs and Salen-Co(III) in MIP systems to improve the conductivity and catalytic activity in the electrochemical oxidation process, demonstrating that the prepared electrode has good stability and sensitivity in methimazole determination (linear range: 0.5–6.0 mg L^−1^; LOD: 0.048 mg L^−1^).

### 2.3. Graphene (GR) and Its Derivatives

GR is a two-dimensional carbon material with a honeycomb lattice structure closely packed by single-layer carbon atoms [21,22]. Its discovery disproved the prediction that isolated two-dimensional crystals could not truly exist, thus arousing great concern in the scientific community [23]. The discovery of GR also triggered a new wave of research on carbon materials after CNTs. The C atoms in GR are sp2 hybridized; the hybrid orbital forms the σ bond with the adjacent C atoms to form a regular hexagonal network structure. GR possesses a super highly specific surface area (approximately 2630 m^2^ g^−1^), and the specific capacitance of GR prepared by the chemical method can reach 100–230 F g^−1^ [24,25]. The GR sheet has a fold structure with the superimposition effect between the layers thus forming nanosized holes and pores, which are conducive to the diffusion of an electrolyte. Thus, GR is an ideal electrode material for a supercapacitor [105,106,107]. A flexible GR-based thin film supercapacitor was fabricated using CNT as current collectors and GR as electrodes. Due to the combination of the high capacitance of the thin GR film and the high conductivity of the CNT film, the fabricated devices obtained high energy density (8–14 Wh kg^−1^) and power density (250–450 kW kg^−1^) [108]. GR with good electrical conductivity, unique quantum Hall effect at room temperature, and extremely fast electron mobility is an ideal material for the formation of nanoelectronic devices [109,110]. Cheng et al. reported the enhanced performance of suspended GR-field effect transistors (GR-FETs) in aqueous solutions. Significantly, the transconductance of GR-FETs in the linear operating modes increases by 1.5 and 2 times when the power of low-frequency noise decreases by 12 and 6 times in the case of the hole and electron carriers, respectively [111]. 

On the other hand, GR materials have a relatively complete structure and stable surface, resulting in poor dispersibility and solubility. Additionally, a strong Van der Waals force between the layers of GR may predispose it to agglomeration, thus inhibiting the widespread use of this type of materials [112,113,114]. Therefore, various inorganic and organic materials or polymers have been used to improve the properties of GR in sensing applications. These GR composite materials have various properties and play various roles in the construction of new food safety sensors [115,116]. Inorganic nanomaterials can be dispersed on the surface of a GR sheet to obtain GR-inorganic nanocomposites [117,118,119]. Inorganic NPs can increase the spacing between the layers of GR and reduce the force between the layers to retain the structure and properties of the monolayer GR. This synergistic effect is important for the applications [120,121]. Liu and coworkers examined the influence of two inorganic NPs, namely, SiO_2_ and Al_2_O_3_, on the adsorption of 17 β-estradiol onto GR oxide using batch adsorption experiments [122]. The results demonstrated that the presence of inorganic NPs significantly inhibits adsorption, and increases the time required to reach adsorption equilibrium for the adsorption of an analyte onto GR. Thus, this study provides new insight into the fate and transport of GR and pollutants in natural aquatic environments.

**Figure 5:**
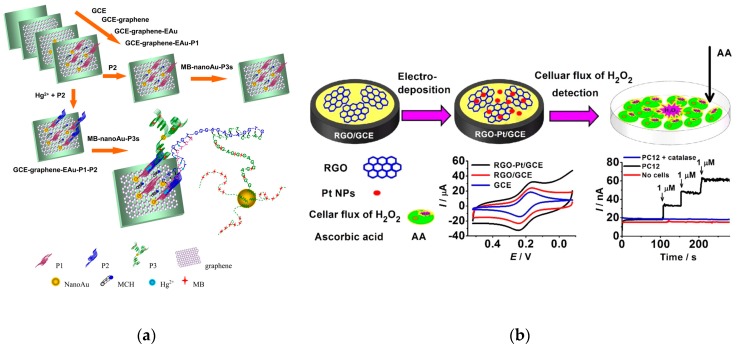
Application of GR and NPs in the fabrication of sensors. (**a**) The construction procedure of GR/AuNPs/GCE for detection of Hg^2+^. Reproduced with permission from reference [128]. Copyright American Chemical Society, 2015. (**b**) GO−Pt nanocomposite-modified GCE for detection of H_2_O_2_ efflux from the cells stimulated with ascorbic acid. Reproduced with permission from reference [130]. Copyright American Chemical Society, 2014.

The large specific surface area of GR makes it an ideal carrier for metal NPs [123,124,125]. The loading of metal NPs onto the surface of graphite sheets avoids the agglomeration of the GR sheets and the NPs; prepared composite materials generally exhibit unique or superior properties. GR/metal NP composites have shown tremendous value in various applications, such as energy, sensor and optoelectronics [126,127]. Zhang’s research group designed three single-stranded DNA probes for Hg^2+^ detection. GR and AuNPs were electrodeposited on the GCE surface to improve the electrode conductivity and functionalize it with the thymine-rich DNA probe. This sensor can detect Hg^2+^ ranging of 1.0 aM–100 nM with LOD of 0.001 aM, demonstrating its feasibility in developing ultrasensitive detection strategies [128,129] (Figure 5a). 

Another study has reported the application of GR-Pt nanocomposites for measuring H_2_O_2_ release from the living cells. Electrochemical study demonstrated that the modified GR-Pt nanocomposites on the GCE surface have a high peak current and low overpotential towards H_2_O_2_ reduction. The sensitivity of the fabricated system was substantially higher than that of the PtNPs-or GR-modified electrodes [130] (Figure 5b). Ma and Chen reduced HAuCl_4_ to AuNPs through cyclic voltammetry on the GR-modified GCE. A good catalytic performance was obtained using GR/AuNPs/GCE for electrochemical oxidation of diethylstilboestrol with good selectivity and stability in food samples [131]. A GR and CNT nanocomposite was directly reduced onto the screen-printed electrode and electrochemically deposited AuNPs for bisphenol A detection in aqueous solution [132].

**Figure 6:**
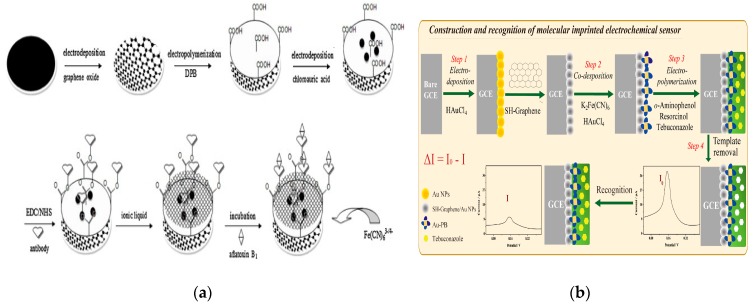
Application of GR combined with ionic liquid in electrochemical sensors. (**a**) GR/conductive polymer/AuNPs/ionic liquid membrane sensor for aflatoxin B_1_ detection. Reproduced with permission from reference [147]. Copyright 2012 Elsevier. (**b**) The preparation procedure for MIP/Au-PB/SH-G/AuNPs/GCE. Reproduced with permission from reference [149]. Copyright 2018 Elsevier.

GR can form more stable composite materials with PDA [133,134], polychitosan [135], polyallylamine [136,137] and other polymers [138,139], thus combining the excellent performance of GR and polymeric materials for extensive applications in food safety [140,141]. Zhang et al. successfully applied the synthesized AgNPs to functionalize PDA-GR nanosheets (AgNPs-PDA-GNS) with uniform and high dispersion. The PDA layer was used as a nanoscale guide to form a uniform AgNPs-PDA-GNS surface. The resultant AgNPs-PDA-GR hybrid material was demonstrated to have strong antibacterial properties against gram-negative and gram-positive bacteria due to the synergistic effect of GR nanosheets and AgNPs [142]. Wang et al. were the first to prepare the poly (sodium 4-styrenesulfonate) (PSS)-functionalized GR through simple one-step reduction of exfoliated GR in the presence of PSS. The isopropanol-nafion-PSS-GR composite-modified GCE has superior electrocatalytic activity towards the oxidation of clenbuterol and was successfully applied for clenbuterol determination in pork [143]. The GR/inorganic/organic nanocomposites can fully utilize the synergistic effect of various materials and have better performance further expanding the application of GR [144,145,146]. Zhou and coworkers electrodeposited the composite membrane of GR/conductive polymer/AuNPs/ionic liquid onto the electrode surface to achieve good stability; GR and AuNPs can ensure an efficient rate of electron transfer. This fabricated electrode was applied for aflatoxin B_1_ detection achieving LOD of 1 fmol L^−1^, concentration range of 3.2 fmol L^−1^–0.32 pmol L^−1^, and recovery of 96.3–101.2% in food samples [147] (Figure 6a). Nitrogen-doped GR with dispersed CuNPs was successfully prepared by one-pot synthesis and applied to construct an amperometric nonenzymatic sensor of glucose with high selectivity and reproducibility and acceptable recovery in complex foods [148].

The combination of GR with MIPs and ionic liquids further enhances the performance of molecularly imprinted biomimetic sensors and expands their applications in food safety [150]. A new molecularly imprinted electrochemical sensor for carbofuran detection was constructed by decorating reduced GR oxide and AuNPs (rGO@AuNPs), which has high adsorption capacity and good selectivity in the detection process of vegetable samples [151]. Thiol GR and AuNPs were introduced to increase the specific surface area to enhance the signal of a probe (PB-AuNPs) immobilized molecularly imprinted electrochemical sensor for selective detection of tebuconazole in vegetable and fruit samples [149] (Figure 6b). Room-temperature ionic liquids are highly conductive and stable and have good solubility in several inorganic salts and organic substances; they are widely used in electrochemistry and organic synthesis [152,153,154]. Zhao et al. were the first to develop a MIP-ionic liquid-GR composite film of methyl parathion. The ionic liquid-modified GR oxide was electrochemically reduced and MIP suspension followed. The developed sensor displayed high selectivity and stability in determination of methyl parathion in the samples (recovery: 97–110%, LOD: 6 nM) [155]. For the determination of carbaryl, an imprinted poly (p-aminothiophenol) (p-ATP) film sensor was constructed with chitosan-AuPt alloy NPs and GR-ionic liquid-Au with Fe(CN)_6_^3−^/Fe(CN)_6_^4−^ as electrochemical probe. The chitosan-AuPtNPs and GR-ionic liquid-Au composites were responsible for immobilization of p-ATP monomer and improvement of electrochemical response [156]. 

The outstanding fluorescence of GR quantum dots (GR-QDs) is an important property [157,158,159]. Currently, GR-QDs that emit fluorescence at various wavelengths can be prepared by controlling the experimental conditions. Compared with traditional QDs, GR-QDs are chemically inert and have low toxicity, good biocompatibility, water solubility photo-bleaching, unique structure and excellent GR characteristics [160,161]. The surface of GR-QDs usually contains oxygen-containing groups, such as –OH and –COOH, which are beneficial to further functional applications. Therefore, GR-QDs are of great potential value in biological imaging, drug targeting transportation, sensors, photoelectrocatalysis, electroluminescence and other areas [162,163,164]. Wang et al. developed a fluorescent method for ochratoxin A (OTA) detection using iron-doped porous carbon and aptamer-functionalized nitrogen-doped GR-QDs as the probes, which can detect concentrations of OTA in the range of 10–5000 nM with LOD of 2.28 nM [165]. Gondim et al. developed an electrochemical method based on an assembly of GR-QDs for the detection of sulfonamide residues, which demonstrated to have a significant increase in detection sensitivity [166]. A sensitive electrochemical sensor based on GR-QDs/riboflavin was constructed and utilized for the determination of persulfate (S_2_O_8_^2−^). The electron transfer coefficient (α) and the heterogeneous electron transfer rate constant (*K*s) for riboflavin redox reaction on GR-QDs/riboflavin-modified GCE achieved 0.52 and 6.59 s^−1^, respectively. This material exhibited an excellent electrocatalytic activity for S_2_O_8_^2−^ reduction with LOD of 0.2 μM, concentration calibration range from 1.0 μM to 1 mM and sensitivity of 4.7 nA μM^−1^ [167]. 

### 2.4. Carbon Dots (CDs)

Fluorescent carbon NPs or QDs (CDs) are a new class of carbon nanomaterials that have emerged recently and have attracted considerable interest as competitors to conventional semiconductor QDs [26,168,169]. In addition to comparable optical properties, desired advantages of CDs have desired advantages of low toxicity, environmental friendliness, low cost and simple synthetic routes. The surface passivation and functionalization of CDs also allow their physicochemical properties to be controlled [27,170,171,172]. These characteristics have led to numerous applications of CDs in the areas of chemo- and biosensing, bioimaging, photocatalysis and electrocatalysis [173,174,175,176]. 

**Figure 7:**
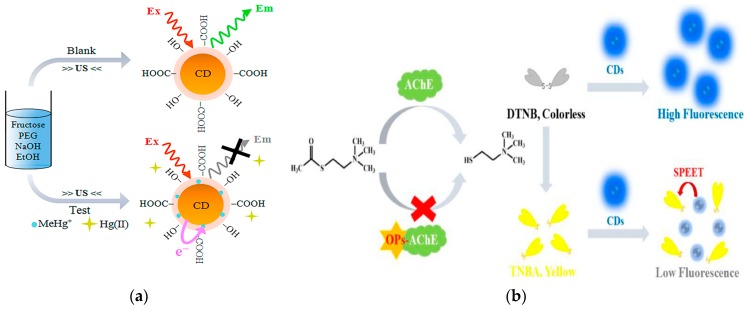

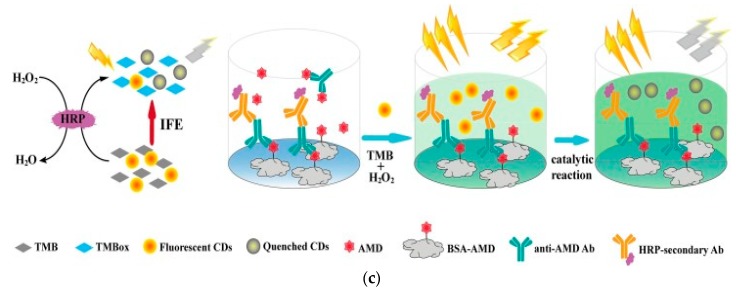
Application of CDs fluorescence quenching for the detection of harmful substances. (**a**) The mechanism involved in the CD fluorescence quenching for methylmercury detection. Reproduced with permission from reference [177]. Copyright 2014 American Chemical Society. (**b**) The principle of inner filter effect-based fluorescence quenching of CDs. Reproduced with permission from reference [178]. Copyright Elsevier, 2018. (**c**) Scheme of the CD-based fluorescent ELISA for amantadine detection. Reproduced with permission from reference [179]. Copyright Elsevier, 2019.

Costas-Mora et al. have reported the ultrasound-assisted synthesis of CDs and its application as optical nanoprobe in the detection of methylmercury [177] (Figure 7a). The application of high-intensity sonication achieves simultaneous the synthesis for fluorescent CD and the selective recognition of the target methylmercury. The assay can be finished within 1 min, with a LOD of 5.9 nM and repeatability expressed as RSD of 2.2% (*n* = 7). Li et al. designed a label-free bioplatform for organophosphorous pesticide (OP) detection through dual-mode (fluorometric and colorimetric) channels based on AChE-controlled quenching of CD fluorescence [178] (Figure 7b). This dual-output assay has good sensitivity, with a LOD of 0.4 ng mL^−1^ (paraoxon), potentially indicating a promising candidate for OP detection. Wang et al. synthesized fluorescent CDs and used them as the signal probes in conventional ELISA to improve the sensitivity. In this strategy, the enzymatically formed products of HRP/alkaline phosphatase efficiently quench the fluorescence of CDs. In the application of detection of residual amantadine in chicken muscle, this fluorescent immunoassay obtains a LOD of 0.02 ng mL^−1^ [179] (Figure 7c). 

**Figure 8:**
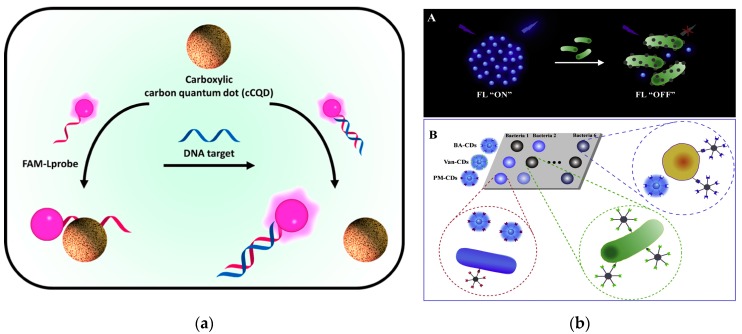
Functionalized CDs for fluorescence detection. (**a**) Schematic illustration of the carboxylic carbon quantum dot (CQD)-based fluorescent detection of DNA. Reproduced with permission from reference [182]. Copyright 2016 American Chemical Society. (**b**) Schematic illustration of pattern recognition of bacteria based on three different receptor-functionalized CDs. Reproduced with permission from reference [183]. Copyright Elsevier, 2019.

The core of the quantum-sized CDs includes carbon atoms stabilized by proper ligands. The main obstacle to development of CD-based sensing devices is fixing CDs in a suitable matrix to maintain their properties and to ensure effective penetration of the analyte while preventing CDs from leaching [26,180,181]. The carboxylic CDs functionalized with citric acid and malic acid were reported to be applied as a nanoquencher for nucleic acids detection in a homogeneous fluorescent assay. For these two types of CDs, a superior detection range of at least 3 orders of magnitude was achieved. These findings provided a valuable insight into the use of CQD in the fabrication of future DNA biosensors [182] (Figure 8a). 

The identification and quantitative analysis of bacteria is a crucial issue in food safety. Conventional methods require long culture time, highly skilled operators, or specific recognition elements to each type of bacteria. The sensor arrays offer a rapid, cost-effective and simple approach using multiple cross-reactive receptors. Facile construction of a fluorescence sensing array based on CDs functionalized with different receptors was reported for identification of various bacteria. Three types of receptors (boronic acid, polymixin and vancomycin) yielded CDs that are able to bind to various bacteria due to variable physicochemical nature of various bacterial surfaces [183] (Figure 8b).

**Figure 9:**
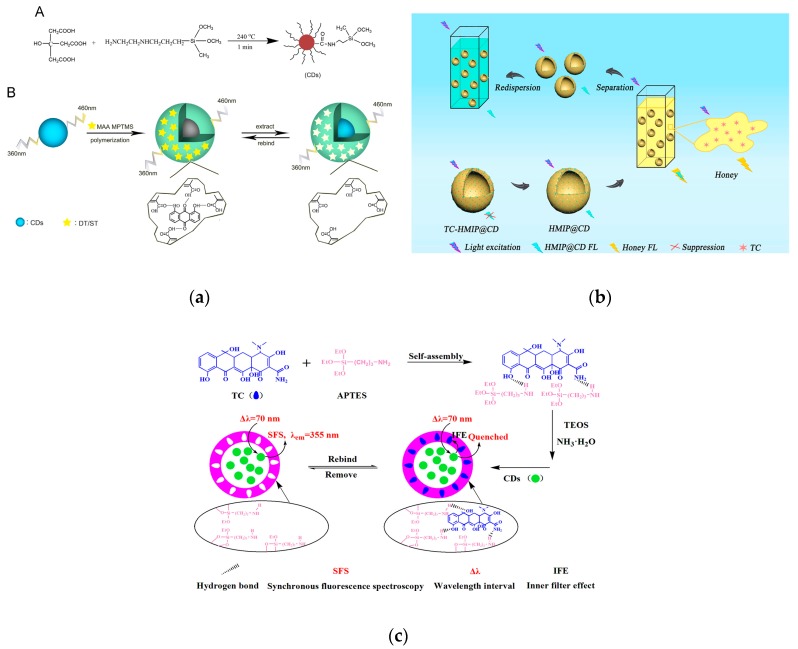
Application of MIP@CD sensor in fluorescence detection. (**a**) Scheme of preparation procedure of CDs@MIP material. Reproduced with permission from reference [184]. Copyright Elsevier, 2016. (**b**) Scheme of the fluorescence detection process of TC in honey. Reproduced with permission from reference [185]. Copyright Elsevier, 2018. (**c**) Schematic diagram of the preparation of MIP@CDs and the identification mechanism of IFE quenching. Reproduced with permission from reference [186]. Copyright Elsevier, 2018.

CD-embedded MIP materials have become an ideal strategy. Xu et al. were the first to synthesize highly blue luminescent CDs followed by a nonhydrolytic sol-gel process for MIP layer formation on the surface. CDs acted as antennas for signal amplification and optical readout and MIP provided specific target-binding sites. Compared with the non-imprinted polymer, CD@MIP-based assay was demonstrated to have excellent selectivity and sensitivity for sterigmatocystin in grains [184] (Figure 9a). In another case, the quantification of tetracycline (TC) in milk, honey and fish samples was achieved using effective luminescence of CDs and specific adsorption of MIPs [185,186,187] (Figure 8b,c). These CD and MIP-involved assays for food safety have revealed two key points of design of luminescent nanomaterial-based MIPs. Specifically, the intense and stable fluorescence signal should be able to pass through the polymer crosslinking layer and further produce a signal readout through the interaction with the target analyte. Additionally, the sufficient cavities in the imprinting polymers are critical for specific recognition to the targets.

## 3. Conclusions

Various nanoscale carbon-based materials are excellent materials for the construction of the sensors due to their outstanding performances. A considerable number of theoretical and practical studies have been carried out describing the preparation, modification and application of carbon-based nanomaterials in the food testing-related field. Substantial progress has been achieved, thus fully demonstrating the prospects of carbon-based nanomaterials as a new sensor construction material. The development of advanced preparation technology, nanotechnology and sensing technology will lead to more advances in the use of carbon-based nanomaterials in studies of food analysis.

## Figures and Tables

**Table 1:** Comparison of the characteristics of typical carbon-based nanomaterials.

Category	Diameter	Dimension	Parameters	Reference
Carbon nanotubes	0.7–100 nm	one	Thermal conductivity: 3500 W m^−1^ K^−1^ (SWCNT); 3000 W m^−1^ K^−1^ (MWCNT); Young’s modulus: 1 TPa	[13,14,15,16]
Ordered mesoporous carbon	2–50 nm	—	Specific surface area: 500–2500 m^2^ g^−1^; Pore volume: 1.5 cm^3^ g^−1^	[17,18,19,20]
Graphene	—	two	Specific surface area: 2630 m^2^ g^−1^; Specific capacitance: 100–230 F g^−1^; Carrier mobility: 15,000 cm^2^ v^−1^·s^−1^; Thermal conductivity: 5300 W m^−1^ K^−1^ (Single layer); Young’s modulus: 1 TPa (theoretical); Resistivity: 10^−6^ Ω·cm	[14,15,21,22,23,24,25]
Carbon dots	<10 nm	zero	—	[26,27]
